# Visual hallucinosis during hypoperfusion of the right occipito-temporal cortex

**DOI:** 10.1007/s00415-022-11346-x

**Published:** 2022-08-24

**Authors:** Nicolae Sanda, Jose Bernardo Escribano Paredes, Victor Ferastraoaru

**Affiliations:** 1 Department of Neurology, Geneva University Hospital, Gabrielle-Perret-Gentil 4, 1205 Geneva, Switzerland; 2 Department of Neurology, Montefiore Medical Center/Albert Einstein College of Medicine, New York, USA

**Keywords:** Visual hallucinations, Cerebral hypoperfusion, Hallucination semiology

## Abstract

**Objectives:**

Positive visual phenomena, although reported in lesions of visual cortex, are often overlooked in patients with acute neurological conditions. Yet, their occurrence without structural abnormalities or other underlying neurological disorders represents a unique observation. This report aims to raise awareness of these phenomena, their implications for understanding visual consciousness and to propose a practical, structured algorithm for the clinical assessment of visual hallucinations related to neurological conditions.

**Methods:**

We describe the clinical presentation and imaging findings in two patients with isolated visual hallucinosis secondary to transitory hypoperfusion.

**Results:**

One patient presented with subocclusion of the right posterior cerebral artery and the other with multifocal arterial abnormalities suggestive of reversible cerebral vasoconstriction syndrome (RCVS). Both presented isolated visual hallucinations and hypoperfusion of the right mesial occipito-temporal cortex. Hallucinated images exhibited peculiarities of certain attributes that were recognized only through guided perceptual analysis performed during their occurrence.

**Discussion:**

Dysfunctions in the visual and attentional networks due to the uneven impact of hypoperfusion on the regions of the mesial occipito-temporal cortex likely contributed to the occurrence of visual hallucinations. The initial impaired awareness of certain image attributes obscured an altered, non-realistic rendering of the hallucinated images. Enhancement of awareness through clinical guidance indicates improved attentional deployment, modulation of visual information processing and hallucination–background integration. These features of the hallucinatory phenomena highlight the critical role of semiological analysis during their occurrence and question the validity of post hoc inquiries.

## Introduction

Visual sensory deprivation (e.g. Charles Bonnet syndrome) and several brain disorders, including strokes in visual areas, may induce hallucinations [1–5]. Their underlying mechanisms remain unclear due to several factors. Firstly, these phenomena are particularly complex and likely entail various dysfunctions of specific brain regions and their connections (hodotopic dysfunctions) [6–8]. Secondly, the transient and dynamic nature of visual hallucinations makes them difficult to explore, many of the available studies being conducted at a distance from symptom occurrence [5, 9]. Finally, the dogma of localization in clinical neurology focuses on perceptual deficits, negative phenomena that are easier to qualify and quantify, and much less on positive phenomena, which are more subjective and more difficult to assess.

We report two cases with visual hallucinosis and hypoperfusion of the right occipito-temporal cortex, without infarction of the visual cortex, and we propose a practical and structured algorithm for the evaluation of visual hallucinations related to neurological conditions. This algorithm allows an organized exploration of the hallucinatory phenomena and the localization of the involved brain networks. It includes the classification of the hallucinations, the context of their appearance, their chronology, the assessment of insight and of the attributes of the hallucinated images (see Table 1). Since it explores dynamic, often transient, perceptual phenomena, guided analysis is particularly useful when performed while hallucinations are actually occurring and much less so when performed at a distance.

**Table 1:** Algorithm for semiological analysis of visual hallucinations

Visual hallucinations		Description	Significance/key points
Type	Simple	Phosphenes, lines, geometric forms, dendropsias (branching shapes), leaves, splashes of colors	Simple hallucinations have origin in the primary cortical visual areas (V1–V4)
	Complex	Biomorphic (e.g. humans, animal, plants), objects, writings, abstract complex forms	Complex hallucinations point to an origin in higher order visual areas
Context and Chronology	Onset	Rapid/slow progression from simple to complex hallucinations; co-occurrence of simple and complex; other related symptoms	Recording of speed progression from simple to complex hallucinations can help estimate the fluctuations in the visual brain function (diaschisis progression from lower to higher-order visual cortex) [10]
	Occurrence conditions	Ambient luminance (low, high); sensory context (effect of other stimuli—e.g., sound, touch—on the visual hallucinations)	Low ambient luminance and suppression by other stimuli (e.g. high luminance, unexpected sound, pinch) indicate a release mechanism
			High ambient luminance and facilitation by other sensory conditions indicate a synesthetic mechanism [26]
	Frequency and duration	Variations in hallucination frequency since occurrence, duration of each episode and the total duration of symptoms	Duration might translate increased functional connectivity between ventral-visual stream and salience network, while increased frequency, decreased functional connectivity between default mode and salience network [25]
	Visual field	Upon steady fixation note in which part of the visual field hallucinations occur (unilateral, bilateral, central) or if they occur outside the visual field (e.g., extracampine hallucinations, autoscopic phenomena)	Hallucinations usually manifest in the hemi-visual field opposite the affected hemisphere; bilateral occurrence may indicate bi-hemispheric disruptions; hallucinations outside the field of view suggest dysfunctions of the temporo-parietal junction [27]
Insight		Record and quantify insight during the entire duration of the hallucinatory phenomena (e.g., full/some/no insight)	In elderly patients, low/ no hallucination-specific insight may point to an associated neurodegenerative disorder [28]
Attributes	Appearance	Record patient’s impression regarding the perception of the hallucinated image—natural appearance, abnormal/dysmorphic features, sketch-like appearance	Abnormal features, sketch-like appearance, abnormal size suggest dysfunction in the lateral fusiform gyrus and occipital cortex coding for face features [29, 30]
	Size/Constancy	Normal size, smaller or larger than normal in relation to the expected size in the point where the hallucination projects into the visual scene. Noteworthy, according to Emmert's law the perceived image size is influenced by the point where hallucination is projected (e.g. small when projected near and large when projected far away)	If the perceived size is influenced by the point where the image is projected, this may translate a normal visual constancy mechanism
			If the hallucination is projected at an explicit distance and its perceived relative-size is abnormal, this may indicate dysfunctions in the occipito-parietal and occipito-temporal networks [29, 30]
	Sharpness	Very well defined, blurry, writings—legible/illegible	Various patterns of dysfunctions in cortical and subcortical structures
	Color	Hyperchromatic (vivid); hypochromatic (pastel); black and white; appropriate or inappropriate color for the image (e.g., a bear with a brown vs pink fur)	Probable disruptions in visual areas and networks involved in color perception (BA18, 19, V8) [9]
	Movement	Present/absent; if present, note whether it occurs during steady fixation or if it is produced by eye movements (follows gaze movement); note if the movement is ecological–biological (walking, flying, moving leaves in the wind), mechanical (the movement of the wheels of a car/bicycle); rotational (around its own axis) or translational (around a reference point)	Simple rotational or translational motion is relatively frequent in the hallucinated images
			Hallucinations fixed in retinotopic coordinates may be perceived in translational motion during eye movements
			Complex motion, especially biological movement requires intricate processing and is uncommon
	Background integration	Hallucinations are superimposed and intermingled with the real scene Note if there is perfect integration/blurred transition/gap between the hallucinated images and background	Hallucinations–background transition often presents alterations that go unnoticed at first
			Perceptual integration of transitional alterations helps patients better cope with hallucinatory phenomena and may be related to the modulation of figure–background segregation processes [22, 23]
	Familiarity	Note whether the hallucinated object or person are known (e.g., family member, personal object)	Hallucinations of known persons or objects suggest the involvement of visual memory networks (hippocampus, parahippocampal gyrus, BA 11/47/45) [9]
	Emotional impact	Distinguish between primary affective component, intrinsically related to hallucinations (neutral/pleasant/unpleasant) and secondary emotional component related to the patient's emotions about the possible implications of the hallucinations (e.g., fear of mental illness)	The presence of a non-neutral primary emotional impact indicates the participation of affective networks (amygdala, BA 11,47,45) [9]
			Non-neutral secondary emotional impact requires reassurance and patient support [3]
Awareness of attributes flaws		Record the baseline, spontaneous level of awareness about flaws in the hallucinated images, immediately after and remote from the guided semiological analysis	Awareness is often altered; its improvement may require guided semiological analysis that might translate modulations in the attentional deployment [24, 25]

## Methods

### Case 1

**Fig. 1:**
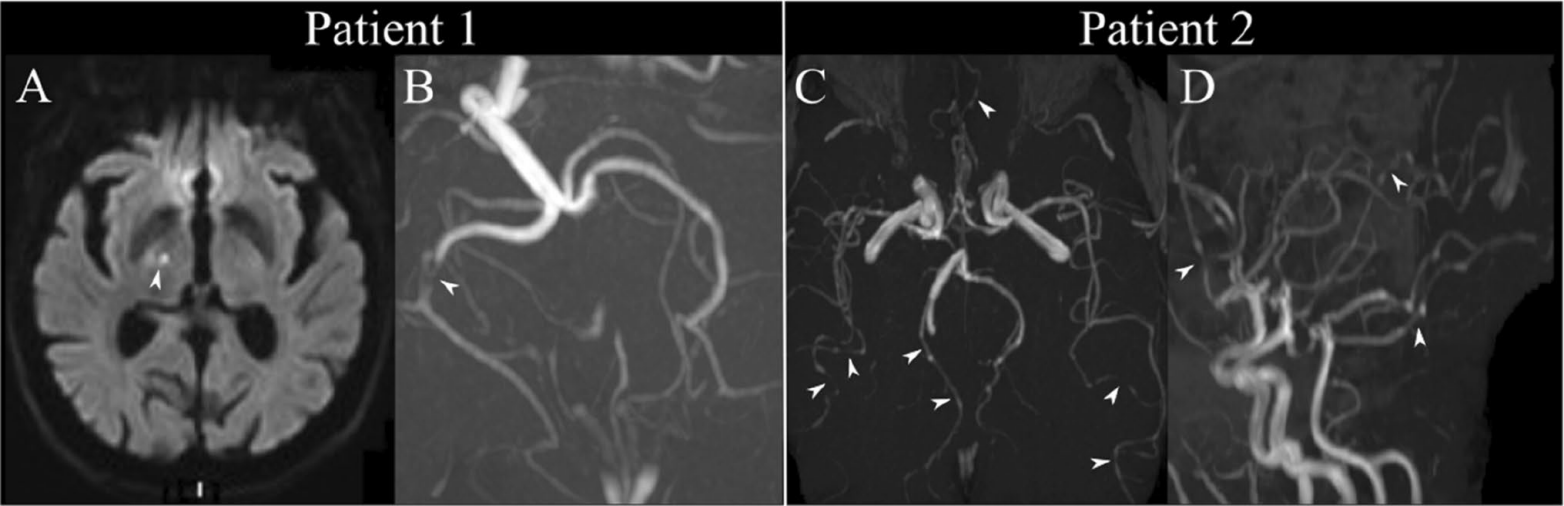
Patient 1 (left): **A** hyperintensity in the right ventro-lateral thalamus on the diffusion weighted imaging (DWI) sequence corresponding to a punctiform acute ischemic lesion and **B** significant stenosis of the P2 segment of the right posterior cerebral artery on the time-of-flight (TOF) sequence (white arrow). Patient 2 (right): **C, D** multiple arterial irregularities (white arrows) on the TOF sequence suggestive of reversible cerebral vasoconstriction syndrome

An 85-year-old woman was admitted following transient left hemianopia, manifesting as a “dark veil on the left” that lasted about 10 h. Her medical history included high blood pressure, atrial fibrillation, breast cancer at age 51 and contralateral recurrence at age 78, pulmonary embolism, and bilateral cataract surgery 4 years ago. Her neurological exam was normal. MRI revealed a punctiform area of restricted diffusion in the right ventro-lateral thalamus (motor relay region) and subocclusion of the P2 segment of the right posterior cerebral artery (Fig. 1A, B).

**Fig. 2:**
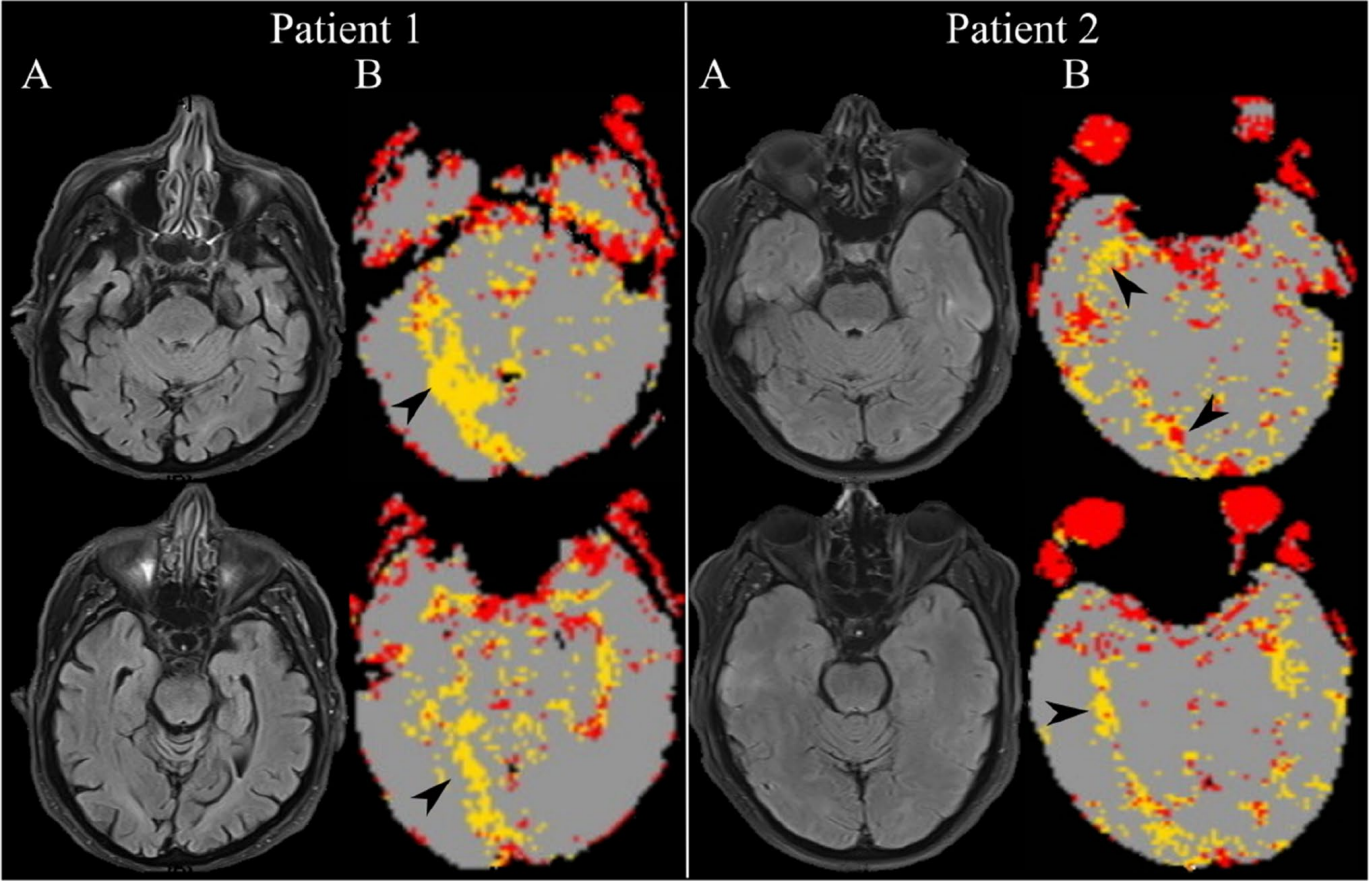
Patient 1 (left) and Patient 2 (right): **A** fluid attenuated inversion recovery (FLAIR) and **B** the corresponding perfusion-weighted imaging (PWI) maps (Olea Sphere®, Olea Medical) showing hypoperfusion in the right mesial occipito-temporal cortex in both patients (black arrows)

The following day, while free of other neurological symptoms, she experienced isolated simple and complex visual hallucinations, more often in darkness or dim light. Insight was preserved. A repeat brain MRI revealed hypoperfusion in the right occipito-temporal cortex without new infarction (Fig. 2A, B). EEG during hallucinations, ophthalmological examination and automated perimetry were normal. The hallucinations gradually decreased in frequency ceasing 3 days later. Repeat brain MRI several days later showed resolution of the P2 segment subocclusion.

Patient's hallucinations were assessed shortly after their first appearance, when they were manifest, and the evolution of the phenomenology was followed daily until its resolution. Simple hallucinations consisted of colored splashes and branching shapes (dendropsias). Her complex hallucinations were clear wall writings, people and plants smaller than real (micropsia), clear and brightly colored. All had neutral emotional impact. Through guided analysis, patient noticed that all hallucinations manifested in her left visual field. The writings were illegible and appeared static. The people and the plants showed depth and lateral shifting without biological movement, resulting in an ellipsoid trajectory, and the transition between the hallucinations and the background was blurred. Noteworthy, lateral component of the translational movement was present during steady fixation and was also influenced by the eye movements (e.g. changes in the perceived trajectory or speed).

### Case 2

A 55-year-old woman with history of untreated essential tremor was admitted following a third episode of thunderclap headache. Neurological exam was normal. MRI showed multifocal arterial irregularities suggestive of RCVS (Fig. 1C, D). No precipitating factors were identified and nimodipine was started.

Several hours later, on the evening of the admission day, patient reported isolated simple and complex visual hallucinations with preserved insight. A repeat MRI showed hypoperfusion of the right occipito-temporal cortex without parenchymal lesions (Fig. 2A, B). EEG during hallucinations, ophthalmological exam and automated perimetry were normal. Hallucinations gradually decreased in frequency, ceasing after 2 days. Repeat MRI at one-month follow-up showed resolution of the segmental arterial constrictions, confirming the RCVS, and absent parenchymal lesions.

Patient's hallucinations were assessed soon after their first appearance, when they were manifest, and their progress was monitored daily until they resolved. Simple visual hallucinations included a bright equilateral triangle sliding from the left towards the center of the visual field, replaced several hours later by complex hallucinations of people, animals and plants. Her complex hallucinations were normal-sized, clear, vividly colored, well integrated into background, “moving” and emotionally neutral, except for one reported as a “scary head with holes in its face, large red cheeks and small black eyes”. Through guided visual analysis, patient noted that all hallucinations manifested in the left visual field and that hallucinations of people, animals and plants had in-depth and lateral shifting without biological movement resulting in an ellipsoid trajectory, and a blurred transition with the background. As with the first patient, the lateral component of translational motion was present during steady fixation and was further impacted by eye movements (e.g. changes in the perceived trajectory or speed).

## Discussion

The occurrence of visual hallucinations during cerebral hypoperfusion in the ventral visual stream without infarction or underlying neurodegenerative disorder suggests dysfunctions in the visual networks [10, 11]. This is an interesting, and to our knowledge, a previously unreported phenomenon.

While in the first patient, simple and complex hallucinations were intermingled in the course of the hallucinatory phenomena, in the second patient there was a progression from simple to complex hallucinations. Insight was preserved and the hallucinations were spontaneously perceived as real and natural. To emphasize the veracity of the perceived images, patients focused on aspects like the high resolution of certain parts of the image (e.g., animal fur, leafs veins) while disregarding others (e.g., motion patterns, legibility of writing) and the guided analysis performed during the phenomenology pointed out peculiarities of certain attributes (see Table 1).

In our patients, hypoperfusion could have induced an aura-like, cortical spreading depression phenomenon similar to the one described in the penumbra region of ischemic stroke [12–14]. Moreover, hypoperfusion likely had an unequal impact on different components of the mesial occipito-temporal cortex inducing variable degrees of dysfunction in the visual hubs. Indeed, the V1 region is more sensitive to hypoxia due to higher cellular density and increased metabolic demand [15]. Therefore, abnormalities of the primary visual cortex may alter the output to higher-order visual areas allowing their spontaneous activation and elaboration of hallucinatory percepts [16]. Hallucinations content reflects the functional specializations of the involved brain regions [16–18], and since the regions encoding faces and objects have a right-hemisphere lateralization, this might explain why both reported patients showed hypoperfusion of the right occipitotemporal cortex. It is possible that the text hallucinations in Case 1 originated in areas of the right occipitotemporal cortex involved in the processing of font and handwriting [19, 20], and that guided analysis revealed their lack of semantic content through recruitment of the visual word form area and language-related areas of the left hemisphere [21].

The change in the perception of the hallucinations following the guided analysis could be due to several reasons. One possibility is that the hallucinations are intrinsically defective and that the analysis helped the patients become aware of the image flaws. Another possibility is that the hallucinations are inherently real when the patient focuses on them but their perception is altered by the guided redeployment of attention towards a global picture integrating the hallucinations into the environment. Indeed, this last hypothesis is endorsed by the fact that most of the flaws detected in the hallucinated images concerned attributes that interact or are impacted by the visual context, such as the type of movement, the relative size and the interface with the background. In physiological conditions, the detection of image boundaries is paramount in figure–background segregation [22], the segregated images being routed to the lateral occipital cortex and the background to more dorsal occipital areas [23]. Conversely, the detection of boundaries in the already segregated, hallucinated images might prompt their integration into the background and help identify flawed attributes. As altered communication between the canonical attentional control networks seems to be involved in the hallucinatory phenomena regardless of their origin [24, 25], guided analysis may therefore optimize the attentional deployment and, therefore, modulate the visual information processing and hallucination–background integration.

## Conclusion

Hallucinated images, although spontaneously reported as “completely natural”, presented structural and kinetic anomalies revealing an altered, non-realistic rendering. Noteworthy, the impaired awareness of peculiarities in the attributes of the hallucinated images questions the accuracy of post hoc inquiries and points to the crucial role of detailed, structured semiological analysis in the acute phase, while visual hallucinations are actually occurring. This should be considered by future studies exploring positive perceptual phenomena.
